# Complete Genome Sequences of Seven New Chrysodeixis includens Nucleopolyhedrovirus Isolates from Minas Gerais and Mato Grosso States in Brazil

**DOI:** 10.1128/MRA.01501-19

**Published:** 2020-02-20

**Authors:** Saluana R. Craveiro, Peter W. Inglis, Laura Lisieux S. Monteiro, Luis Arthur V. M. Santos, Roberto C. Togawa, Zilda Maria A. Ribeiro, Bergmann M. Ribeiro, Maria Elita B. Castro

**Affiliations:** a Embrapa Recursos Genéticos e Biotecnologia, Brasília, Federal District, Brazil; b Departamento de Biologia Celular, Universidade de Brasília-UnB, Brasília, Federal District, Brazil; c Centro Universitário de Brasília–UniCEUB, Brasília, Federal District, Brazil; DOE Joint Genome Institute

## Abstract

We report the complete genomic sequences of seven viral isolates from the soybean looper (Chrysodeixis includens) from midwestern and southeastern Brazil. The genomes range from 138,760 to 139,637 bp in length with a G+C content of 39.2% and 140 open reading frames (ORFs).

## ANNOUNCEMENT

Chrysodeixis includens nucleopolyhedrovirus (ChinNPV) is a group II *Alphabaculovirus* of the family *Baculoviridae* that has Pseudoplusia includens single nucleopolyhedrovirus-IE (PsinNPV-IE; GenBank accession number KJ631622) as its representative isolate (1, 2). We report here the complete genome sequences of seven new isolates of this species, originally obtained from infected *Chrysodeixis includens* (Walker) (Lepidoptera: Noctuidae) larvae collected on soybeans and cotton in the states of Minas Gerais and Mato Grosso, Brazil. The isolates were selected for sequencing due to their differences in virulence, attractive properties that will benefit the development of biopesticides. Virus isolates consisted of purified occlusion bodies (OBs) as described previously (3). Samples from these isolates are deposited in the EMBRAPA Collection of Invertebrate Viruses and are listed in the Brazilian AleloMicro Information System (http://alelomicro.cenargen.embrapa.br/). DNA was purified from OBs of each viral isolate using the DNeasy blood and tissue kit (Qiagen). The genomes were sequenced using the Illumina Nextera XT DNA library preparation kit and the MiSeq reagent kit v2 nano 250-nucleotide (nt) paired-end platform (Table 1). Data were assembled with default parameters in all cases using IVA 1.0.3 (4), IDBA 1.1.2 (5), VICUNA 1.3 (6), and SPAdes 3.10.1 (7). Final genome assembly and comparative analyses were performed in Geneious R9 9.1.8 (8). Open reading frames (ORFs), which were annotated if encoded by 50 or more amino acids and were initiated with a methionine codon, were predicted in Geneious using the ORFfinder module (NCBI). Genome circularity was implied by reads overlapping across the contig edges, as expected based on baculovirus DNA genomes, and by convention, the *polh* gene was considered the first gene.

**TABLE 1:** Assembly and availability of ChinNPV genome sequences

Viral isolate (AleloMicro accession code)	GenBank accession no.	No. of raw data sequences	No. of sequences after filtering	Coverage^*a*^ (×)	Genome size (bp)	G+C content (%)	SRA accession no.	DNA identity with standard isolate PsinNPV-IE (%)
ChinNPV-MG.A (BRM 050760)	MN542939	412,286	411,820	689	139,470	39.2	SRX7298277	99.45
ChinNPV-MG.B (BRM 050761)	MN542938	528,980	528,432	885	139,637	39.2	SRX7298278	99.14
ChinNPV-MT.A (BRM 028529)	MN689112	453,794	452,056	772	139,113	39.2	SRX7298279	99.23
ChinNPV-MT.B (BRM 028530)	MN689113	500,240	498,556	849	139,074	39.2	SRX7298280	99.20
ChinNPV-MT.C (BRM 050757)	MN689114	447,588	445,936	764	138,760	39.2	SRX7298281	99.06
ChinNPV-MT.D (BRM 050758)	MN689115	465,804	464,546	798	139,046	39.2	SRX7298282	99.14
ChinNPV-MT.E (BRM 050759)	MN689116	539,766	539,156	905	139,225	39.2	SRX7298283	99.24

a Assuming a 139-kb genome size.

A total of 140 ORFs were annotated in each genome, including all baculovirus core genes, but like other previously sequenced ChinNPV genomes (1, 9; NCBI taxonomic identifier 1207438), no typical baculovirus homologous repeat (hr) regions were found in any of the isolates. No major differences in gene content and order were found compared to all known ChinNPV genomes, other than the ChinNPV#1 isolate, which was already identified as distinct in a previous study (9). The genomes of the seven isolates analyzed here exhibited two copies of the *p26* gene, two *bro* (baculovirus repeated ORF) genes (*bro-a* and *bro-b*), one copy of DNA photolyase, and a lack of a *lef-12* homolog. Unlike PsinNPV-IE (1) and ChinNPV#1 (9), which possess two copies of the *he65* gene, the newly sequenced ChinNPV genomes have only one copy. The genome sequence identity between PsinNPV-IE (1) and the new isolates varied from 99.06 to 99.45% (Table 1).

These genomes have high sequence similarity, despite their different geographical origins. However, our previous studies on pathogenicity showed significant variation in their insecticidal properties (3; L. A. V. M. Santos, unpublished data), which we speculate is due to single-nucleotide polymorphisms (SNPs) among the isolates, which are present in ORFs and control sequences. Our findings on the ChinNPV-MG and ChinNPV-MT genomes and those from the ChinNPV-IA to ChinNPV-IG series of isolates, also reported by our group (1, 10), will improve our understanding of the evolution and functional genomics of the baculoviruses.

### Data availability.

The GenBank accession numbers are given in Table 1. Raw sequence data were submitted to the SRA under BioProject number PRJNA594711.
